# Diagnostic compatibility of various fixed orthodontic retainers for head/neck MRI and dental MRI

**DOI:** 10.1007/s00784-023-04861-2

**Published:** 2023-01-14

**Authors:** Alexander Juerchott, Christoph J. Roser, Muhammad Abdullah Saleem, Mathias Nittka, Christopher J. Lux, Sabine Heiland, Martin Bendszus, Tim Hilgenfeld

**Affiliations:** 1 Department of Neuroradiology, Heidelberg University Hospital, Heidelberg, Germany; 2 Department of Orthodontics and Dentofacial Orthopedics, Heidelberg University Hospital, Heidelberg, Germany; 3 Division of Experimental Radiology, Department of Neuroradiology, Heidelberg University Hospital, Heidelberg, Germany; 4 Siemens Healthineers, Erlangen, Germany

**Keywords:** Diagnostic imaging, Magnetic resonance imaging, Dentistry, Orthodontics, Orthodontic retainers, Artifacts

## Abstract

**Objectives:**

To evaluate the diagnostic MRI compatibility of different fixed orthodontic retainers using a high-resolution 3D-sequence optimized for artifact reduction.

**Materials and methods:**

Maxillary and mandibular retainers made of five different materials were scanned in vitro and in vivo at 3 T MRI using an MSVAT-SPACE sequence. In vitro, artifact volumes were determined for all maxillary and mandibular retainers (AV_max_; AV_mand_). In vivo, two independent observers quantified the extent of artifacts based on the visibility of 124 dental and non-dental landmarks using a five-point rating scale (1 = excellent, 2 = good, 3 = acceptable, 4 = poor, 5 = not visible).

**Results:**

Rectangular-steel retainers caused the largest artifacts (AV_max_/AV_mand_: 18,060/15,879 mm^3^) and considerable diagnostic impairment in vivo (mean landmark visibility score ± SD inside/outside the retainer areas: 4.8 ± 0.8/2.9 ± 1.6). Smaller, but diagnostically relevant artifacts were observed for twistflex steel retainers (437/6317 mm^3^, 3.1 ± 1.7/1.3 ± 0.7). All retainers made of precious-alloy materials produced only very small artifact volumes (titanium grade 1: 70/46 mm^3^, titanium grade 5: 47/35 mm^3^, gold: 23/21 mm^3^) without any impact on image quality in vivo (each retainer: visibility scores of 1.0 ± 0.0 for all landmarks inside and outside the retainer areas).

**Conclusions:**

In contrast to steel retainers, titanium and gold retainers are fully compatible for both head/neck and dental MRI when using MSVAT-SPACE.

**Clinical relevance:**

This study demonstrates that titanium and gold retainers do not impair the diagnostic quality of head/neck and dental MRI when applying an appropriate artifact-reduction technique. Steel retainers, however, are not suitable for dental MRI and can severely impair image quality in head/neck MRI of the oral cavity.

## Introduction

Magnetic resonance imaging (MRI) plays a central role in radiology and its use has continued to increase substantially in recent years, including head and neck imaging [1, 2]. Furthermore, dental MRI is becoming increasingly important in different specialties of dentistry, including orthodontics [3–5], periodontology [6–8], endodontics [9–12], and implantology [13–15]. This trend is enhanced by recent technical advances [16–19].

However, the diagnostic benefits of head/neck and dental MRI can be severely compromised by metal-induced susceptibility artifacts originating from orthodontic appliances [20–26]. In this context, MRI artifacts caused by orthodontic retainers are of particular importance, as the number of patients permanently wearing fixed retainers continues to increase [27–29]. Therefore, detailed knowledge of artifacts caused by different retainer materials is crucial for patient care and provides an essential basis for both orthodontists and radiologists in clinical routine. So far, however, there is insufficient data available on MRI artifacts caused by retainers, as previous in vivo studies were mainly based on conventional two-dimensional (2D) sequences and only a maximum of two different retainers were included [21, 22, 25, 26, 30, 31].

As diagnostic limitations caused by retainer-associated MRI artifacts are becoming increasingly relevant in clinical routine, it is of great importance to implement modern MRI techniques that have proven to efficiently reduce metal-artifacts and to compare the extent of artifacts for different retainer materials. The present study for the first time used an MSVAT-SPACE (multiple slab acquisition with view angle tilting gradient based on a sampling perfection with application optimized contrasts using different flip angle evolution) sequence for the evaluation of retainer-associated artifacts. This sequence has proven to efficiently reduce metal-artifacts caused by different dental-materials [19, 32] and allows for high-resolution three-dimensional (3D) imaging of the craniomaxillofacial area [4]. MSVAT-SPACE was applied to quantify artifacts caused by five different retainer materials in vitro and in vivo. The selection of retainers was made based on widely used alloy compositions: two highly paramagnetic steel retainers and three retainers with low paramagnetic properties (titanium grade 1, titanium grade 5, and a gold-platin alloy) were included.

The aim of this combined in vitro and in vivo study was to evaluate the diagnostic MRI compatibility of different fixed orthodontic retainers using a high-resolution 3D-sequence optimized for artifact reduction.

## Materials and methods

### Study participant and ethics

For in vivo MRI, a 32-year-old male participant without contraindications to MRI was enrolled the study. The study was approved by the institutional ethics committee of the University of Heidelberg (approval number: S-452/2010), and written informed consent was obtained.

### Retainers

Five common retainer types were included. For each retainer type, maxillary and mandibular alginate impressions were taken from the volunteer to create plaster models from super-hard dental stone (Hinrizit, Ernst Heinrichs GmbH, Goslar, Germany). All maxillary and mandibular canine-to-canine retainers were bent on these plaster models and then used for both in vitro and in vivo examinations. The weight of each retainer was measured to four decimal places using an analytical balance (R180D, Sartorius Research; Goettingen, Germany). The retainer volume (RV) was determined by dividing the mass by the density. The relevant information on alloy components, RV, and retainer dimensions is given in Table 1.

**Table 1:** Retainer materials used in the study

Product name (reference number)	Manufacturer	Material composition	Volume of maxillary retainer	Volume of mandibular retainer
Remanium ideal arch, rectangular (767–103-01)	Dentaurum, Ispringen, Germany	Stainless steel alloy 304	6.52 mm^3^	4.98 mm^3^
Respond archwire, 5-strand twisted (264–1121)	Ormco, Orange, CA, USA	Stainless steel alloy 304	7.01 mm^3^	5.17 mm^3^
Titanium retainer wire grade 1, 3-strand twisted (528–000-01)	Dentaurum, Ispringen, Germany	Titanium grade 1	6.23 mm^3^	4.72 mm^3^
Titanium retainer wire grade 5, 3-strand twisted (528–000-00)	Dentaurum, Ispringen, Germany	Titanium grade 5	6.18 mm^3^	4.62 mm^3^
Gold retainer, 3-strand twisted (529–000-00)	Dentaurum, Ispringen, Germany	Gold-platinum alloy	5.55 mm^3^	4.25 mm^3^

### MRI examinations

In vitro and in vivo MRI scans were performed on a 3 Tesla MRI system (MAGNETOM Trio, Siemens Healthineers, Erlangen, Germany). A 3D T1-weighted MSVAT-SPACE prototype sequence was used for both in vivo and in vitro MRI examinations. This sequence was specifically optimized and evaluated for 3D MRI of the craniofacial area [19, 33]. Sequence parameters were echo time: 5.8 ms, repetition time: 800 ms, bandwidth: 625 Hz/pixel, number of averages: 1, echo train length: 100, field of view: 171 mm × 171 mm, acquisition matrix: 320 × 320, voxel size: 0.53 mm × 0.53 mm × 0.53 mm, number of sections: 256, and time of acquisition: 7:01 min.

For in vitro scans, a 16-channel multi-purpose coil (Variety, Noras MRI products GmbH, Hoechberg, Germany) was used, with retainers embedded in agar gel (Select Agar, ThermoFisher Scientific, Waltham, MA, USA) as described elsewhere [34].

For in vivo scans, maxillary and mandibular acrylic splints (Duran, Scheu Dental, Iserlohn, Germany) were fabricated for the volunteer. All in vivo examinations were performed in centric occlusion with lips and tongue in a resting position using a dedicated 15-channel dental coil (Mandibula, Noras MRI products GmbH, Hoechberg, Germany). MR images were taken (1) with empty splints to verify that the splints did not cause any artifacts and (2) with retainers attached to the splints, according to previous studies [20, 25, 35]. For each retainer material, both maxillary and mandibular retainers were inserted.

### In vitro quantification of artifact volumes

Artifact volumes (AV) for all investigated maxillary and mandibular retainers (AV_max_; AV_mand_) were quantified by a standardized semi-automated segmentation procedure [36] using Amira software (Version 6.4.0, ThermoFisher Scientific, Waltham, MA, USA). This workflow allowed for separate 3D identification of signal loss and pile-up artifacts. AV was obtained by adding signal loss and pile-up artifact volumes and subtracting the RV.

### In vivo assessment of artifacts

All in vivo datasets were assessed by two independent observers (AJ and TH, both radiologists with seven years’ experience in head/neck and dental MRI). The extent of in vivo artifacts was quantified by assessing the visibility of pre-defined anatomic landmarks using an established five-point rating scale [37, 38]. The visibility of all landmarks was graded as follows: 1 = excellent, 2 = good, 3 = acceptable, 4 = poor, and 5 = not visible. For each retainer material, 124 landmarks were assessed, including dental and non-dental (head and neck related) landmarks inside and outside the retainer areas (Table 2).

**Table 2:** Anatomic landmarks used for in vivo assessment of retainer artifacts

Landmarks in areas with retainer	Landmarks in areas without retainer
Dental (36 landmarks): incisal edge, pulp chamber and apical foramen of all teeth with retainer (13–23 and 33–43, respectively)	Dental (36 landmarks): incisal edge, pulp chamber, and apical foramen from first premolar to first molar in each quadrant (14–16, 24–26, 34–36, and 44–46, respectively)
Non-dental (32 landmarks): alveolar limb of all teeth with retainer (13–23 and 33–43, respectively), anterior nasal spine, incisive foramen, point A (maximum midline concavity on the maxilla), point B (maximum midline concavity on the mandibula), menton, anterolateral edge of the tongue left/right, tip of the tongue, anterior part of genioglossus muscle left/right, anterior part of sublingual gland left/right, anterior part of the palatal masticatory mucosa left/right, inner side of the lower lip, inner side of the upper lip, anterior part of the upper alveolar mucosa left/right, and anterior part of the lower alveolar mucosa left/right	Non-dental (20 landmarks): alveolar limb from first premolar to first molar in each quadrant (14–16, 24–26, 34–36, and 44–46, respectively), posterolateral edge of the tongue left/right, posterior part of the palatal masticatory mucosa left/right, posterior part of the upper alveolar mucosa left/right, and posterior part of the lower alveolar mucosa left/right

### Statistical analysis

For in vitro data, the ratio of artifact volume to retainer volume was calculated for both upper and lower jaw retainers (AV/RV_max_; AV/RV_mand_). For in vivo analyses, weighted kappa (κ) with quadratic weights and the 95% confidence interval (CI) were calculated using SPSS Version 27 (SPSS Inc., Chicago, IL, USA) to determine the inter-rater reliability of landmark visibility scoring. For each retainer type, mean values and standard deviations (SD) of visibility scores were calculated for landmarks in areas with retainer and landmarks in areas without retainer. Furthermore, landmarks in areas with retainer were subdivided into upper jaw landmarks, lower jaw landmarks, incisal edges, pulp chambers, apical foramina, and non-dental landmarks.

## Results

### In vitro quantification of artifact volumes

The largest artifacts were observed for the rectangular steel retainer, with values of 18,060 mm^3^ for AV_max_ (proportion of pile-up artifacts: 3.1%) and 15,879 mm^3^ for AV_mand_ (pile-up artifacts: 2.3%). The AV/RV_max_ ratio was 2270, compared to an AV/RV_mand_ ratio of 3189.

For the twistflex steel retainer, AV_max_ was 7437 mm^3^ (pile-up artifacts: 5.0%) and AV_mand_ was 6317 mm^3^ (pile-up artifacts: 2.5%). The corresponding AV/RV_max_ and AV/RV_mand_ ratios were 1061 and 1222, respectively. Thus, artifacts caused by the twistflex steel retainer were considerable, yet substantially smaller than those observed for the conventional rectangular steel retainer.

Retainers made of titanium grade 1, titanium grade 5, and gold generally caused small artifacts. Titanium grade 1 revealed an AV_max_ of 70 mm^3^ (pile-up artifacts: 38.7%) and an AV_mand_ of 46 mm^3^ (pile-up artifacts: 41.1%), with AV/RV_max_ and AV/RV_mand_ ratios of 11 and 10, respectively. For the titanium grade 5 retainer, values of 47 mm^3^ for AV_max_ (pile-up artifacts: 42.7%) and 35 mm^3^ for AV_mand_ (pile-up artifacts: 36.7%) were observed. Corresponding ratios between artifact and retainer volumes were 8 for AV/RV_max_ and 8 for AV/RV_mand_. The gold retainer caused an AV_max_ of 23 mm^3^ (pile-up artifacts: 34.1%), an AV_mand_ of 21 mm^3^ (pile-up artifacts: 28.9%), an AV/RV_max_ ratio of 4, and an AV/RV_mand_ ratio of 5.

**Fig. 1:**
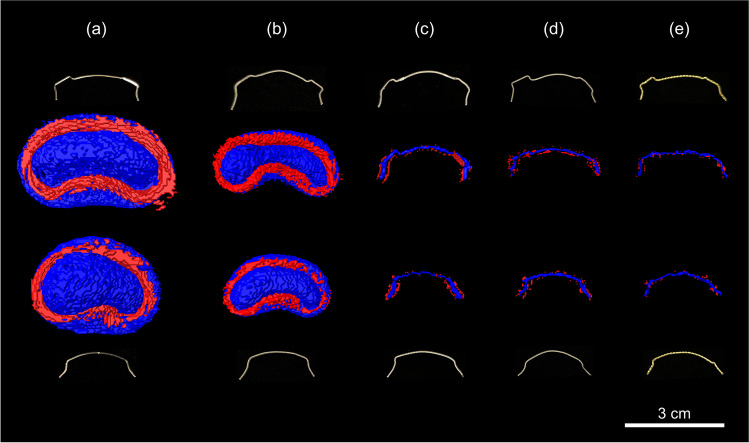
3D volume rendering of artifact volumes of retainers. Rendered artifact volumes (blue: signal-loss artifacts; red: pile-up artifacts) and corresponding photographs are shown for maxillary retainers (upper two rows) and mandibular retainers (lower two rows). (**a**) Rectangular stainless steel. (**b**) Twistflex stainless steel. (**c**) Titanium grade 1. (**d**) Titanium grade 5. (**e**) Gold

**Fig. 2:**
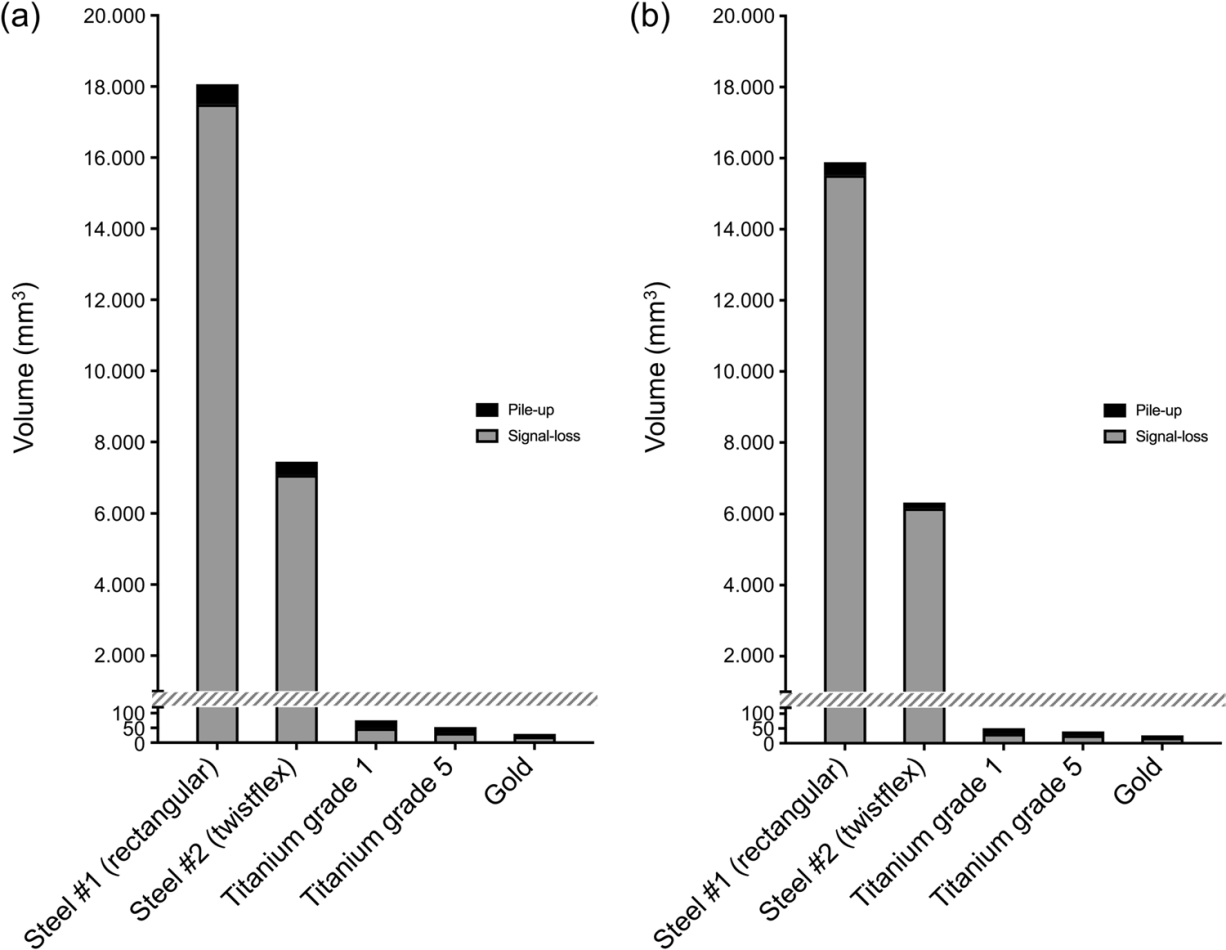
Volume of pile-up and signal-loss artifacts of maxillary (**a**) and mandibular (**b**) retainers

Figures 1 and 2 illustrate the extent of pile-up and signal-loss artifacts caused by all included maxillary and mandibular retainers.

### In vivo assessment of dental and head/neck related landmarks

An excellent inter-rater reliability was observed for landmark visibility scoring of all included retainer materials, with κ-values (95% CI) of 0.954 (0.935–0.973) for rectangular steel, 0.940 (0.916–0.964) for twistflex steel, 1.000 (1.000–1.000) for titanium grade 1, 1.000 (1.000–1.000) for titanium grade 5, and 1.000 (1.000–1.000) for gold.

**Fig. 3:**
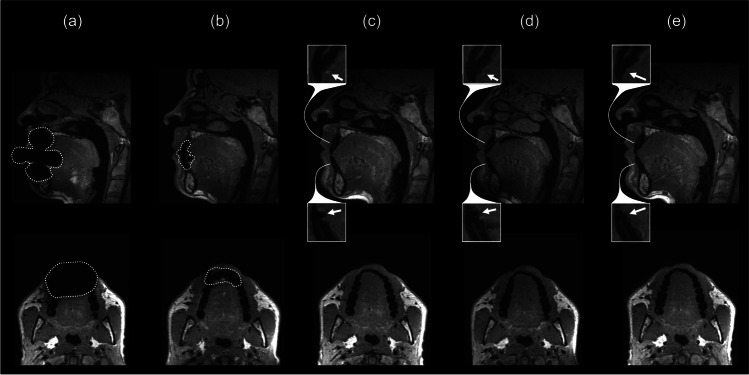
Exemplary in vivo MRI images with different retainers inserted. Images show multi-planar reconstructions in sagittal (upper row) and transverse (lower row) orientation for different retainer materials: (**a**) Rectangular stainless steel. (**b**) Twistflex stainless steel. (**c**) Titanium grade 1. (**d**) Titanium grade 5. (**e**) Gold. The artifact margins of the two steel retainers are highlighted by dotted lines (**a**, **b**). The very small artifacts caused by titanium grade 1, titanium grade 5, and gold are marked by arrows on enlarged image sections (**c**–**e**)

**Fig. 4:**
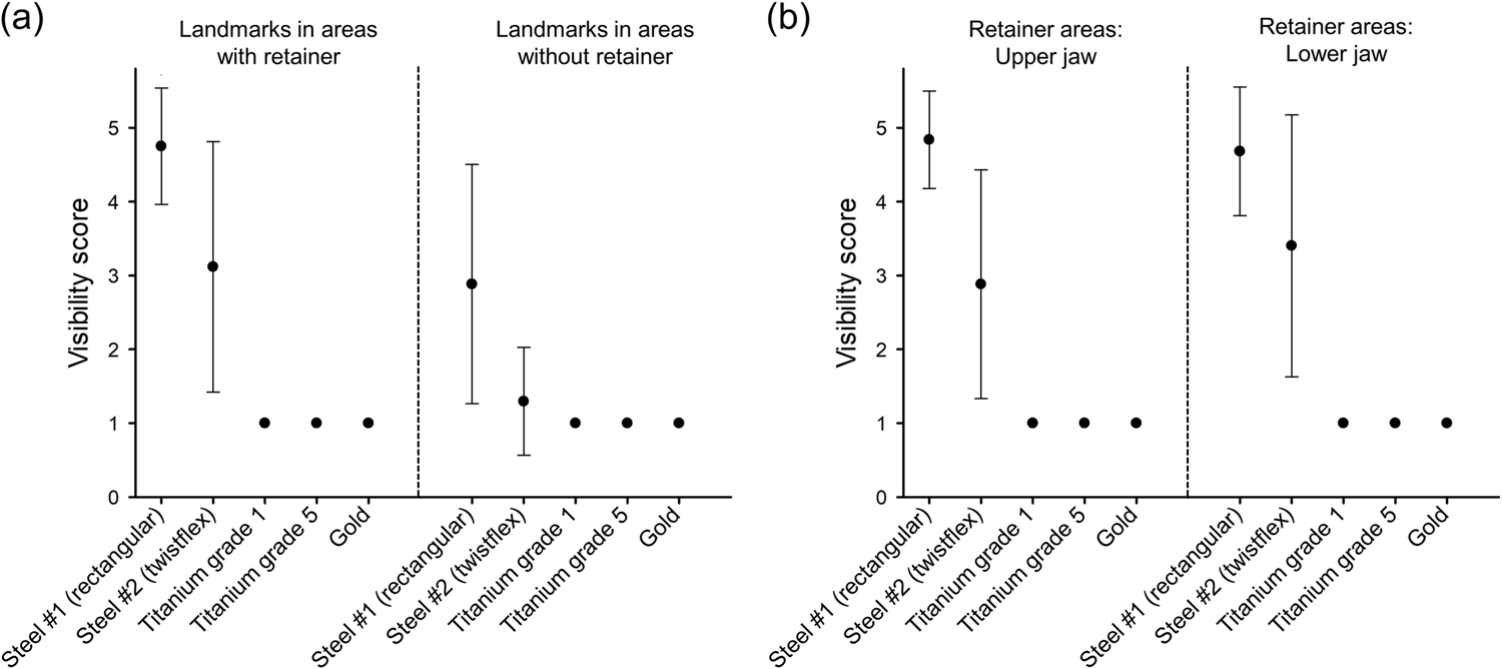
Visibility scores of anatomical landmarks for areas with vs. without retainer (**a**) and for upper vs. lower jaw in retainer areas (**b**). Black dots represent mean values, and thin bars show standard deviations (if applicable)

**Fig. 5:**
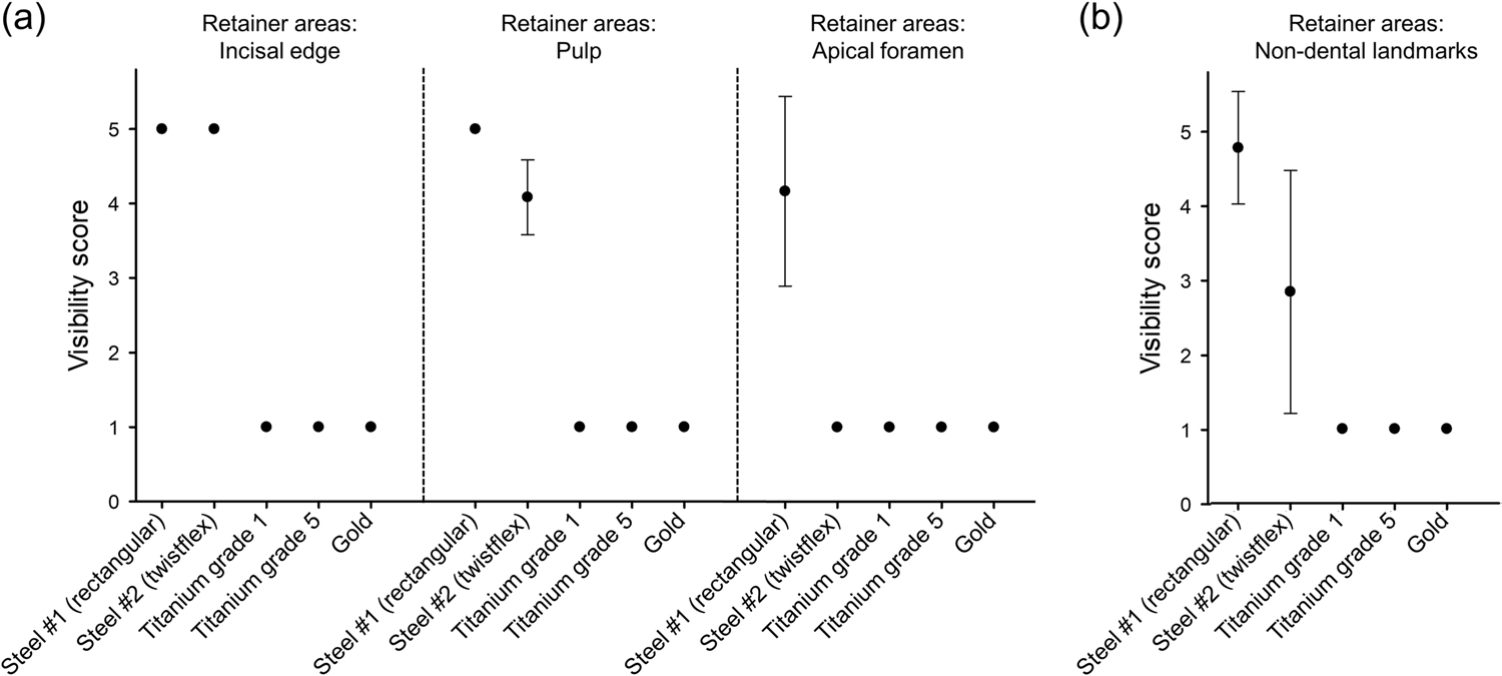
Visibility scores of dental (**a**) and non-dental (**b**) landmarks within retainer areas. Black dots represent mean values, and thin bars show standard deviations (if applicable)

With the rectangular steel retainer inserted, large artifacts were observed in vivo (Fig. 3a) resulting in a strong impact on landmark visibility. The vast majority of predefined anatomical structures within the retainer areas were not visible, leading to a mean visibility score ± SD of 4.8 ± 0.8 (Fig. 4a). For landmarks outside the retainer areas, visibility score was 2.9 ± 1.6 (Fig. 4a). In areas with retainer, subgroup analyses revealed visibility scores of 4.8 ± 0.7 for upper jaw, 4.7 ± 0.9 for lower jaw (Fig. 4b), 5.0 ± 0.0 for incisal edges, 5.0 ± 0.0 for dental pulps, 4.2 ± 1.3 for apical foramina (Fig. 5a), and 4.8 ± 0.8 for non-dental landmarks (Fig. 5b).

The twistflex steel retainer caused smaller in vivo artifacts in comparison to the conventional steel retainer (Fig. 3b). Within the retainer areas, average visibility score was 3.1 ± 1.7 (Fig. 4a). In areas without retainer, only minor diagnostic impairment was observed, leading to a visibility score of 1.3 ± 0.7 (Fig. 4a). Subgroups within the retainer areas showed visibility scores of 2.9 ± 1.6 for upper jaw, 3.4 ± 1.8 for lower jaw (Fig. 4b), 5.0 ± 0.0 for incisal edges, 4.1 ± 0.5 for dental pulps, 1.0 ± 0.0 for apical foramina (Fig. 5a), and 2.8 ± 1.6 for non-dental landmarks (Fig. 5b).

By contrast, retainers made of titanium grade 1, titanium grade 5, and gold did not cause any diagnostic impairment in vivo (Fig. 3c–e). Consequently, an excellent visibility of all included landmarks was achieved for these three retainer materials, with visibility scores of 1.0 ± 0.0 for all included dental and non-dental landmarks within and outside the retainer areas (Figs. 4a and b and 5a and b).

## Discussion

To our knowledge, this study is the first to systematically evaluate MRI artifacts caused by several different orthodontic retainers both in vitro and in vivo using a high-resolution 3D MRI protocol optimized for artifact reduction. Results demonstrate that artifacts associated with titanium and gold retainers are negligible and do not have any influence on image quality in vivo, even directly adjacent to the retainer wires. Accordingly, retainers made of titanium and gold are fully compatible not only for head/neck MRI but also for dental MRI. By contrast, retainers made of stainless steel produce considerable (twistflex-steel) to severe (rectangular-steel) artifacts, even when using an established artifact reduction technique. Consequently, stainless steel retainers are not compatible for dental MRI and can also affect head/neck related landmarks outside the retainer area. These findings are of high clinical relevance, as most fixed orthodontic retainers are still made of stainless steel in today’s orthodontic practice, whereas titanium and gold retainers only account for a relatively small proportion [29, 39–41]. Furthermore, it is important to consider that retainer-associated MRI artifacts will play an even greater role in the future, since the numbers of MRI scans of the craniofacial area and of patients with fixed orthodontic retainers are increasing at the same time [1, 2, 27–29].

The present study has several methodological strengths. First, all in vitro and in vivo MRI scans were performed using state-of-the-art 3 T MRI techniques for head/neck imaging, including dedicated coil systems which have demonstrated high performance in previous studies [12, 18, 19, 33, 42, 43]. Consequently, a high signal-to-noise ratio was achieved allowing for scanning with isotropic voxels and high spatial resolution. The selection of retainer materials is an additional asset of this study: For in vitro and in vivo assessment of retainer-associated MRI artifacts, we chose clinically established retainers made of high-susceptibility materials (two stainless steel retainers) vs. low susceptibility materials (titanium grade 1, titanium grade 5, and gold) to include the “extreme ends” of artifact burdens that may be encountered in clinical routine. Furthermore, the use of a high-resolution 3D sequence offered important benefits for both in vitro and in vivo image analysis: from a clinical point of view, it must be noted that isotropic (3D) sequences are becoming increasingly important in head/neck MRI [44, 45] and also play a key role in dental MRI [6–8, 46–49]. In this context, the MSVAT-SPACE sequence used in this study has demonstrated high diagnostic value in several previous in vivo studies [4, 13, 43, 50]. Another important asset of this isotropic high-resolution sequence is that it enables precise quantification of retainer artifacts. Based on this methodological advantage, it was possible to quantify retainer artifact volumes in vitro by using a recently established, semi-automatic, threshold-based segmentation protocol [36] and to perform detailed 3D analyses of image quality in vivo by including small anatomical structures in direct proximity to the maxillary and mandibular retainers. To our knowledge, the present MRI study is the first to investigate the impact of retainer artifacts on the visibility of landmarks that play a pivotal role in dental MRI.

The capability of the applied MSVAT-SPACE sequence to reduce metal-induced artifacts is reflected by our results, particularly in comparison with our previous in vitro MRI study. In this previous study, MRI artifacts associated with the same twistflex steel retainers were quantified using a conventional SPACE sequence under otherwise identical conditions [34]. In direct comparison of these two studies, MSVAT-SPACE offers a reduction of artifact volumes of 53% for the maxillary twistflex steel retainer (SPACE vs. MSVAT-SPACE: 15,642 vs. 7437 mm^3^ for AV_max_ and 2235 vs. 1061 for AV/RV_max_) and 53% for the mandibular twistflex steel retainer (SPACE vs. MSVAT-SPACE: AV_mand_ 13,530 vs. 6317 mm^3^ for AV_mand_ and 2602 vs. 1222 for AV/RV_mand_). These findings are also consistent with an in vitro study using dental implants in which MSVAT-SPACE revealed a 56% reduction of artifact volumes compared to SPACE [19]. Furthermore, our results are in line with a recent vitro and in vivo study by Sonesson et al. comparing a conventional 2D TSE sequence and a 2D TSE sequence with an included artifact reduction technique (view angle tilting and slice encoding for metal artifact correction) with regard to artifacts produced by a stainless steel retainer [31]. Results revealed considerably smaller artifacts for the TSE sequence optimized for artifact reduction in direct comparison with the conventional TSE sequence. Thus, our findings correspond well with earlier studies and emphasize that MRI sequences enabling an efficient reduction of metal-induced artifacts are of crucial importance in head/neck MRI as well as dental MRI.

Several previous studies have investigated MRI artifacts caused by different orthodontic materials. All these studies used conventional MRI-protocols without specific artifact reduction, except for the above-mentioned study by Sonesson et al. assessing the impact of view angle tilting and slice encoding for metal artifact correction in 2D TSE sequences [31]. Importantly, all previous studies included only one retainer type [22, 25, 26, 30, 31] or at most two different materials [21, 51, 52]. By contrast, the present study systematically compared five different retainer materials in vitro and in vivo using a 3D-SPACE sequence with MSVAT for artifact reduction:

- For both included steel retainers, we found considerable artifact volumes in vitro leading to relevant diagnostic limitations in vivo. In this regard, it should be considered that substantial differences in artifacts between the two stainless steel retainers were observed which may be explained by the manufacturing process resulting in different martensitic properties between retainers of identical stainless steel alloys [51]. Despite these comparatively large differences between the rectangular-steel and the twistflex-steel retainer, our findings are in line with previous analyses on MRI-artifacts caused by steel retainers: Blankenstein et al. found large artifacts caused by a steel retainer on gradient echo and spin echo sequences in vitro [51]. In vitro und in vivo analyses by Tymofiyeva et al. revealed very strong artifacts on gradient echo and spin echo sequences leading to a considerable impairment of image quality in vivo [21]. Shalish et al. assessed a twistflex steel retainer on a dry skull using spin echo sequences and found severe MRI distortions in the regions of the tongue, mandibula, and maxilla [52]. In vivo analyses of Beau et al. based on a spin echo sequence showed massive MRI artifacts produced by a steel retainer that even exceeded the oral cavity [22]. Similarly, Zhylich et al. found non-diagnostic artifact scores in the oral and the pharyngeal regions caused by a steel retainer on spin echo and gradient echo sequences [25]. Finally, Ozawa et al. demonstrated strong MRI artifacts in the anterior oral cavity caused by a steel retainer on fast “cine mode” sequences in vivo [26]
- The two titanium retainers caused only minimal artifacts in the present study, and importantly, these artifacts did not have any influence on image quality in vivo, even when assessing anatomical structures directly adjacent to the retainers (e.g., incisal edges and pulp chambers of teeth in the retainer area). These results are consistent with Blankenstein et al.’s in vitro study revealing no visible MRI artifacts for a titanium retainer scanned on gradient echo and spin echo sequences [51]. Similarly, an in vitro study by Roser et al. using a 3D SPACE sequence found only minimal artifacts caused by CAD/CAM retainers made of nickel-titanium and titanium grade 5 [34]. Moreover, our findings correspond well with in vitro and in vivo data from Tymofiyeva et al. demonstrating very small MRI artifacts caused by a nickel-titanium retainer on gradient echo and spin echo sequences resulting in only slight distortions [21]
- Similar to the two titanium retainers, we found only very small artifact volumes for the gold retainer which also did not affect the diagnostic image quality in vivo. This is in accordance with a skull phantom-based study by Shalish et al. revealing no distortions on MR images acquired with spin echo sequences [52]. Furthermore, an in vivo study by Aizenbud et al. using standard sequences for brain imaging found no visible artifacts in association with a gold retainer [30]

Some limitations have to be considered for this study. Despite the aforementioned methodological strengths, it must be noted that MRI artifacts caused by orthodontic retainers depend on many influencing factors. In this regard, especially the retainer material and the applied MRI technique should be mentioned: although our analyses included more retainer materials than all previous in vitro and in vivo studies, there are further materials that should be investigated in future research. Furthermore, MRI artifacts are influenced by a wide range of factors that are specifically related to the setup of the MRI scanner used, including field strength, sequence properties, and coil systems. Accordingly, taking into account these influencing factors, the results of the present study can only be interpreted within the context of the specific methodological approach. However, it is very likely that our key findings (no diagnostic impairment for titanium and gold retainers vs. diagnostically relevant artifacts for stainless steel retainers) will be confirmed when using different MRI protocols including similar techniques for artifact suppression.

## Conclusions

This study using a high-resolution 3D MSVAT-SPACE sequence optimized for artifact reduction provides important new information on the diagnostic compatibility of different retainer materials for head/neck MRI and dental MRI:

I. Retainers made of gold or titanium produce only minimal artifacts which do not affect the diagnostic quality in both dental and head/neck MRI under clinical conditions
II. Ferromagnetic steel retainers, however, cause relevant MRI artifacts of varying extent, even when applying a robust artifact suppression technique. These retainers severely impair image quality in proximity to orthodontic retainers (rectangular steel and twistflex-steel) and can also affect more distant anatomical structures (rectangular steel)

These findings are of major clinical relevance for orthodontists as well as radiologists and may contribute to an increased use of gold or titanium retainers in the future.                                                                                                                                                                                                                                 

## Data Availability

The datasets generated and analyzed in the current study are available from the corresponding author upon reasonable request.
